# Identification of Novel Plasma Protein Biomarkers for Early Clinical Stage Colorectal Carcinoma by High-Throughput Proteomics

**DOI:** 10.3390/ijms27219630

**Published:** 2026-10-28

**Authors:** Guoxing Yin, Zhifei Chen, Guofeng Feng, Jiangtao Lu, Chenmeng Jiao, Zhengmao Zhu, Lin Liu, Chen Xu

**Affiliations:** 1 State Key Laboratory of Medicinal Chemical Biology, Nankai University, Tianjin 300350, China; 2 Frontiers Science Center for Cell Responses, College of Life Sciences, Nankai University, Tianjin 300071, China; 3 Department of Colorectal Surgery, Tianjin Union Medical Center, Tianjin Institute of Coloproctology, Tianjin 300121, China; 4 Tianjin Union Medical Center, Nankai University, Tianjin 300071, China; 5 Haihe Laboratory of Cell Ecosystem, Tianjin 300020, China

**Keywords:** colorectal carcinoma, proteomics, early clinical stage, machine learning, biomarker

## Abstract

Early detection is critical for improving survival in colorectal carcinoma (CRC), underscoring the need for reliable early-stage biomarkers. Plasma protein biomarkers may support the detection of early-stage CRC. In this study, we profiled plasma from 24 patients with stages I–III CRC and 10 non-tumor participants, together with paired tumor and distant-normal tissues from the patients with CRC, to identify proteins preferentially elevated in stage I disease. Seven plasma proteins had higher abundance in stage I CRC than in stages II and III CRC and non-tumor participants; three of these proteins, HSPA8, ALDOA, and PDLIM1, were also elevated in stage I tumor tissues. In the discovery comparison of stage I CRC with non-tumor participants, HSPA8 alone achieved an internally cross-validated random forest classification accuracy of 88.9%, matching the three-protein model. Tissue upregulation was further supported by Western blotting. ELISA assessment included 32 participants with matched proteomic measurements and was subsequently expanded to 108 participants. The HSPA8-score was calculated by dividing the dilution-corrected HSPA8 concentration by the total protein amount in 10 μL of plasma. In the expanded cohort, random forest classification using this score achieved internally cross-validated accuracies of 74.47% for stage I versus stages II–IV CRC and 56.67% for stage I CRC versus non-tumor participants. The corresponding exploratory reference cutoffs were 0.066 and 0.029. These findings identify HSPA8 as a potential plasma protein biomarker for stage I CRC detection.

## 1. Introduction

Colorectal carcinoma (CRC) remains a major public health burden. An estimated 152,810 new cases and 53,010 deaths were reported in the United States for 2024 [1]. Prognosis depends strongly on disease stage at diagnosis. Although molecularly guided therapies have improved outcomes for selected patients with metastatic CRC, treatment responses remain heterogeneous [2]. These challenges support the development of reliable, minimally invasive biomarkers for early detection and disease monitoring [3]. In this context, liquid biopsy-based approaches and circulating molecular markers have emerged as promising frontiers for improving detection, prognosis, and longitudinal monitoring in CRC patients.

The plasma proteome contains tissue-derived proteins and offers a potential source of biomarkers for disease detection and monitoring [4]. Population-scale proteogenomics studies have linked circulating proteins to genetic variation and disease [5], while cell-selective proteomics in pancreatic cancer models has identified tumor-derived extracellular proteins that reflect cancer subtypes in circulation [6]. In CRC, integrated analyses of plasma proteomic and genetic data have nominated candidate biomarkers and therapeutic targets [7]. Prospective studies and meta-analyses have also linked selected circulating proteins or predefined protein panels to CRC risk [8,9,10,11,12]. However, an association with future CRC risk does not establish diagnostic performance for existing stage I disease. Targeted proteomic platforms provide scalable measurements of predefined protein sets, whereas discovery mass spectrometry (MS) offers complementary coverage without requiring a prespecified list of protein targets. Nevertheless, the large dynamic range of plasma protein concentrations limits the detection of low-abundance proteins [4,13]. Using depletion-assisted deep profiling across five cancer types, Tognetti et al. identified CRC-associated candidates, including STAT3, and found that candidate biomarkers were predominantly drawn from the low-abundance portion of the plasma protome [13]. Plasma protein panels and plasma extracellular-vesicle protein biomarkers have also been investigated for early CRC detection [14,15,16]. Further work is needed to establish their performance in clinically relevant populations, particularly for stage I disease. Here, we integrated stage-stratified plasma proteomics with paired tumor and distant-normal tissue analyses to identify proteins preferentially elevated in stage I CRC. We used random forest classification to prioritize candidates and subsequently assessed HSPA8 by ELISA in an expanded cohort. Public transcriptomic and chromatin datasets were examined to provide biological context.

## 2. Results

### 2.1. Overall Synopsis of Plasma Proteome Profiling of Patients with CRC

The discovery cohort comprised eight patients with stage I CRC, seven with stage II CRC, nine with stage III CRC, and 10 non-tumor participants. Plasma was analyzed from all 34 participants. Tissue proteomics included paired tumor and distant-normal specimens from the 24 patients with CRC and intestinal tissue specimens from four non-tumor participants (Figure S1 and Table S1).

**Figure 1:**
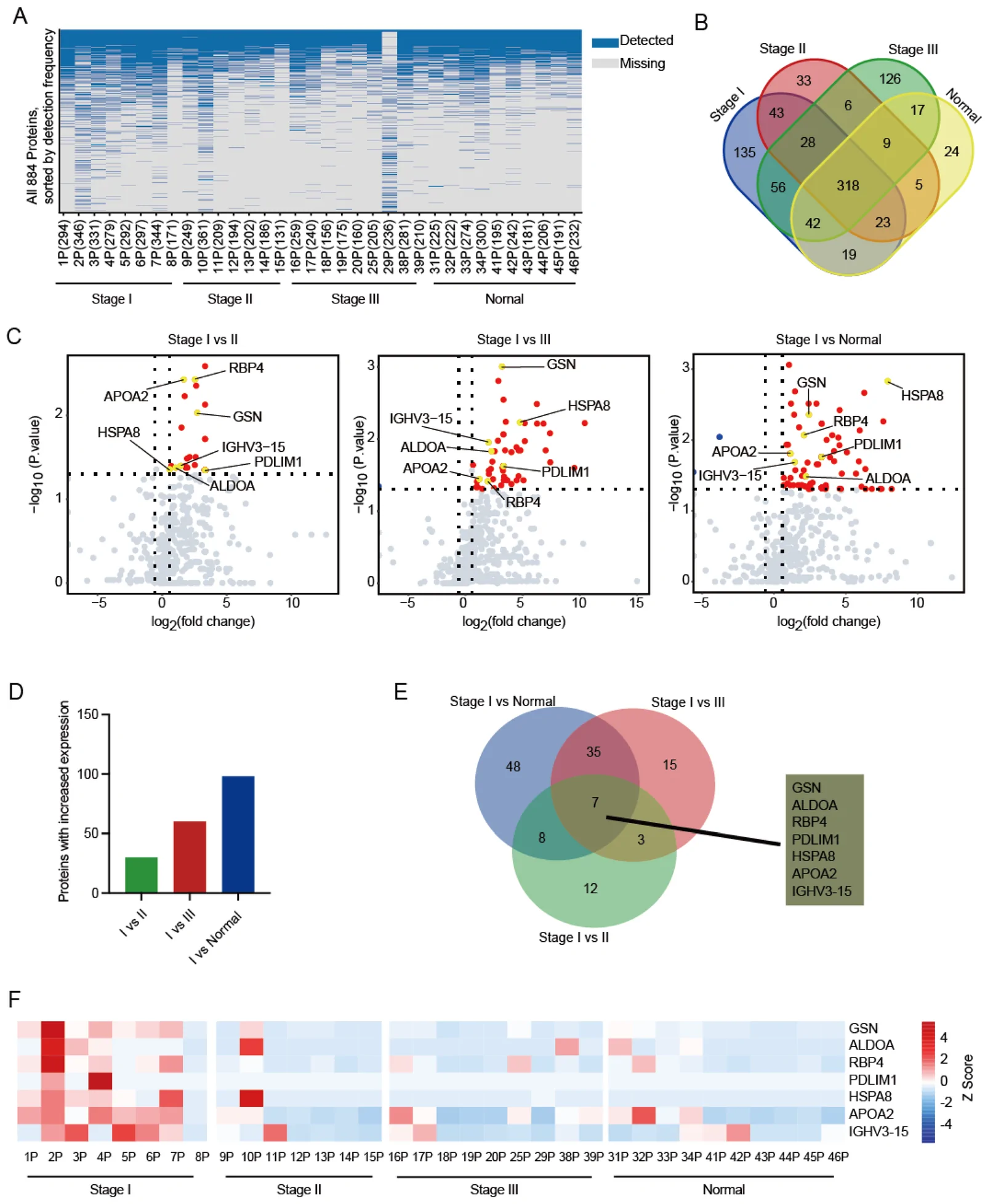
Plasma proteomic profiling across CRC stages. (**A**) Detection heatmap of 884 proteins in plasma from participants with stage I, II, or III CRC and non-tumor participants. Rows represent proteins ordered by detection frequency, and columns represent individual samples. Blue indicates detected proteins; gray indicates missing values. (**B**) Venn diagram showing the overlap of detected plasma proteins among the four groups. (**C**) Volcano plots comparing stage I CRC with stage II CRC, stage III CRC, and non-tumor participants. Red and blue indicate proteins with higher and lower abundance, respectively, in stage I CRC; yellow highlights proteins elevated in all three comparisons. The discovery criteria for elevated proteins were *p* < 0.05 and fold change > 1.5. (**D**) Numbers of proteins with higher abundance in stage I CRC in each comparison. (**E**) Overlap of proteins elevated in stage I CRC across the three comparisons. (**F**) Heatmap of the seven shared candidates across all participant groups, with protein abundances standardized as row-wise z scores. Column labels indicate sample identifiers.

A total of 884 proteins were detected in plasma (Figure 1A and Figure S2A). Of these, 318 (36%) were detected in all four participant groups (Figure 1B). Using *p* < 0.05 and a fold change > 1.5 as the discovery criteria, we identified 30 proteins with higher plasma abundance in stage I than in stage II CRC, 60 with higher abundance in stage I than in stage III CRC, and 98 with higher abundance in stage I CRC than in non-tumor participants (Figure 1C,D and Figures S3–S5). Proteins elevated in stage I relative to stage II CRC were enriched for neutrophil degranulation, whereas those elevated relative to stage III CRC or non-tumor participants were enriched for platelet degranulation (Figure S6). Seven proteins were shared across all three sets of proteins elevated in stage I CRC (Figure 1E). Their plasma abundance patterns are shown in Figure 1F. These proteins were therefore selected for further tissue-level assessment.

### 2.2. Relationship Between Plasma Proteins and Tissue Proteins in Patients with CRC

**Figure 2:**
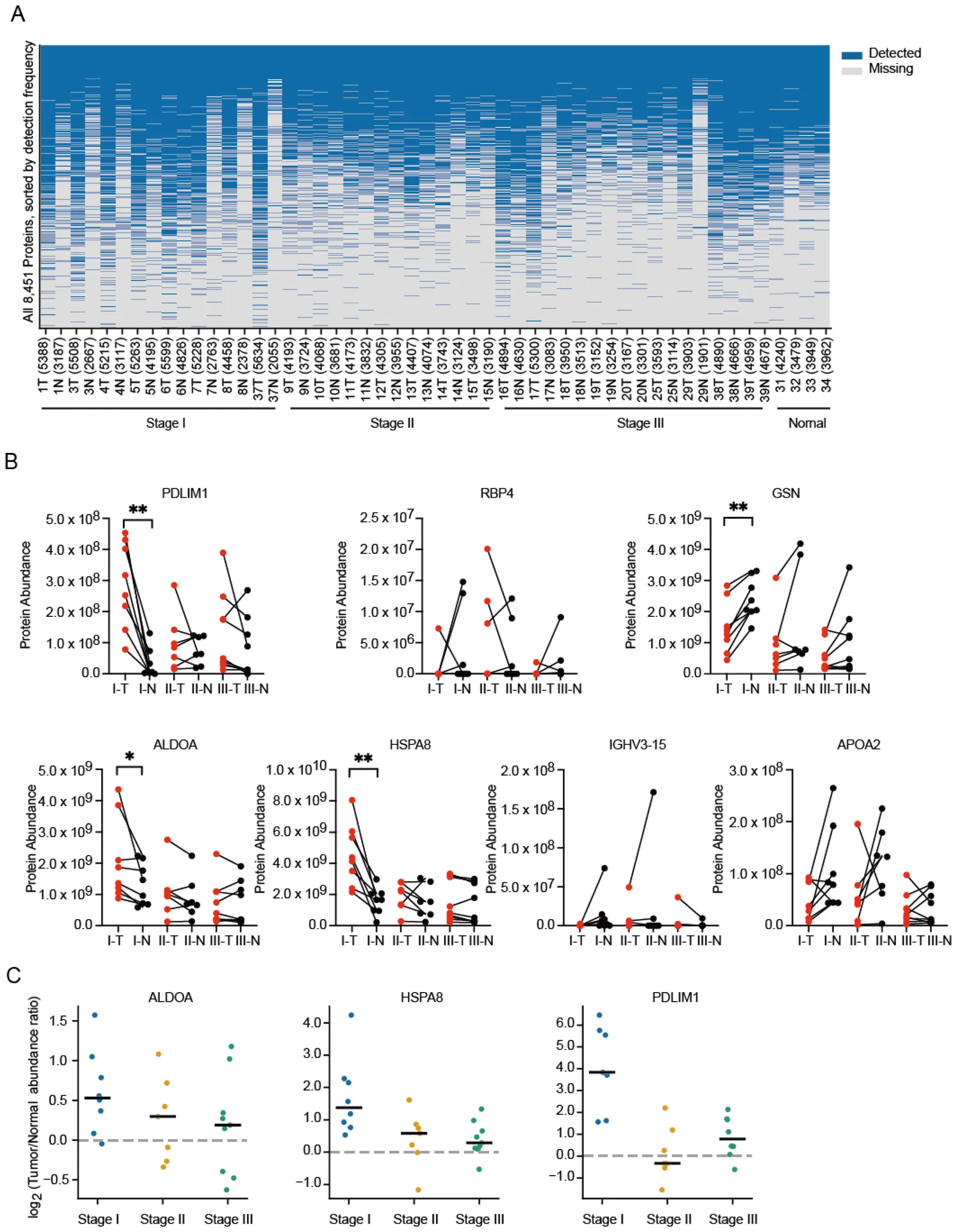
Tissue abundance of plasma protein candidates. (**A**) Detection heatmap of 8451 proteins across tumor (T) and distant-normal (N) tissue pairs from 24 patients with stage I (*n* = 8), stage II (*n* = 7), or stage III (*n* = 9) CRC, together with four non-tumor intestinal specimens. Rows represent proteins ordered by detection frequency, and columns represent individual specimens. Blue indicates detection; gray indicates missing values. Numbers in parentheses indicate the number of proteins detected in each specimen. (**B**) Tissue abundances of plasma candidates in tumor and normal-tissue groups. Bar heights represent raw MS abundance. * *p* < 0.05; ** *p* < 0.01. (**C**) Log_2_-transformed tumor-to-normal abundance ratios for ALDOA, HSPA8, and PDLIM1 by clinical stage. Each point represents one complete tissue pair; black horizontal bars indicate medians.

Tissue proteomics detected 8451 proteins across paired tumor and distant-normal tissues and the four non-tumor intestinal specimens (Figure 2A and Figure S2B). Differences in protein detection between tissue and plasma reflect their distinct biological composition and analytical characteristics; detection alone does not establish a tissue source for a circulating protein. We examined the tissue abundance of the seven plasma candidates. ALDOA, PDLIM1, and HSPA8 showed higher abundance in stage I tumor tissues than in the corresponding distant-normal tissues (Figure 2B). Their tumor-to-normal abundance ratios were also higher in stage I than in stages II and III CRC (Figure 2C). These concordant tissue and plasma patterns supported prioritizing the three proteins for exploratory classification.

Tissue composition may contribute to these abundance patterns. Previous single-cell and spatial studies have identified tumor-enriched SPP1-positive macrophages and FAP-positive fibroblasts in CRC [17], and comparisons with tumor-distant mucosa have shown enrichment of granulocytes and a higher regulatory T-cell fraction in tumors [18]. Moreover, GSN, another plasma candidate, has been reported to be less abundant in colon cancer tissue than in non-tumor colon tissue [19]. Thus, differences between plasma and tissue measurements should be interpreted in the context of cell composition and biological compartment.

### 2.3. Exploratory Classification Prioritizes HSPA8 for ELISA Assessment

**Figure 3:**
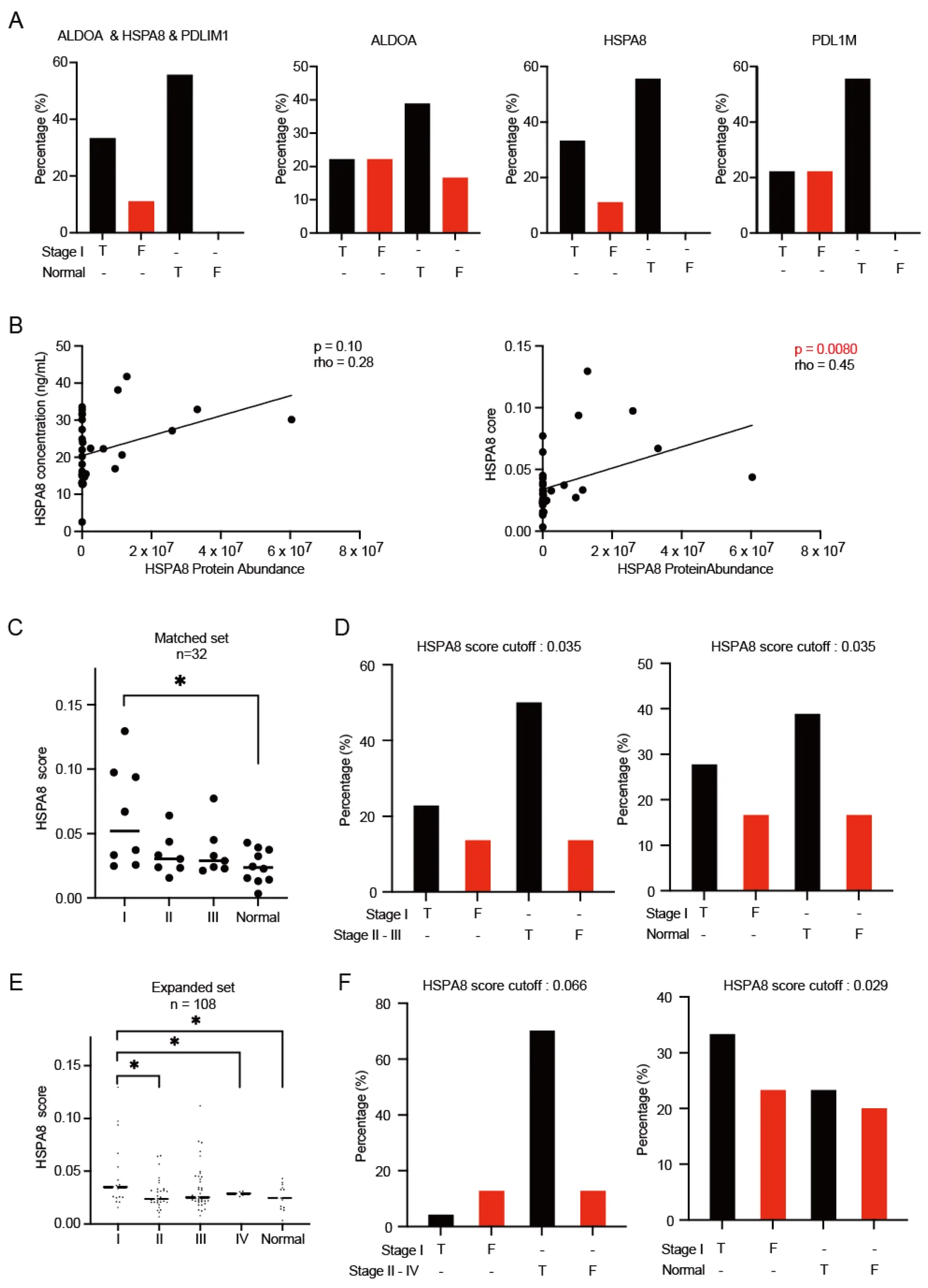
Exploratory classification and ELISA assessment of HSPA8. (**A**) Random forest classification of stage I CRC (*n* = 8) versus non-tumor participants (*n* = 10) using the plasma MS abundances of ALDOA, HSPA8, and PDLIM1 individually or in combination. T and F denote correct and incorrect classifications, respectively; bar heights express counts as percentages of all 18 participants. (**B**) Associations of MS HSPA8 abundance with ELISA-measured HSPA8 concentration (left) and HSPA8-score (right) in 32 matched participants. The score is the dilution-corrected HSPA8 concentration divided by the total protein amount in 10 μL of plasma. Spearman’s *ρ* was 0.28 (*p* = 0.10) for concentration and 0.45 (*p* = 0.0080) for the score. (**C**) HSPA8-score distributions in the matched subset: stage I (*n* = 8), stage II (*n* = 7), stage III (*n* = 7), and normal (*n* = 10); each point represents one participant. * *p* < 0.05 (**D**) Score-based random forest classification in the matched subset: stage I versus stages II–III CRC (*n* = 22; left) and stage I CRC versus non-tumor participants (*n* = 18; right), with accuracies of 72.73% and 66.67%, respectively. (**E**) HSPA8-score distributions in the expanded cohort: stage I (*n* = 16), stage II (*n* = 34), stage III (*n* = 40), stage IV (*n* = 4), and normal (*n* = 14); each point represents one participant. * *p* < 0.05 (**F**) Score-based random forest classification in the expanded cohort: stage I versus stages II–IV CRC (*n* = 94; left) and stage I CRC versus non-tumor participants (*n* = 30; right), with accuracies of 74.47% and 56.67%, respectively. T/F labels and percentage denominators in panels D and F follow the convention in panel A, using the total sample size of each comparison. All classification results are internal estimates from stratified 10-fold cross-validation. The annotated cutoffs in D (0.035 for both comparisons) and F (0.066 and 0.029) were selected post hoc by inspecting observed scores alongside random forest predictions and are not independently validated diagnostic thresholds.

We evaluated the plasma MS abundances of ALDOA, PDLIM1, and HSPA8, individually and in combination, for classifying stage I CRC (*n* = 8) versus non-tumor participants (*n* = 10). Using stratified 10-fold cross-validation, the three-protein random forest model achieved 88.9% accuracy. The corresponding accuracies were 61.11% for ALDOA, 77.78% for PDLIM1, and 88.9% for HSPA8 (Figure 3A). HSPA8 was selected for further assessment because its observed accuracy matched that of the three-protein model. To assess HSPA8 using an antibody-based assay, we performed ELISA in 32 participants with matched plasma proteomic measurements: eight with stage I CRC, seven with stage II CRC, seven with stage III CRC, and 10 non-tumor participants. The association between ELISA-measured HSPA8 concentration and MS abundance was weak and not statistically significant (Spearman’s *ρ* = 0.28, *p* = 0.10; Figure 3B). Because equal total protein inputs were used for MS analysis, we additionally evaluated an HSPA8-score, defined as the dilution-corrected HSPA8 concentration divided by the total protein amount in 10 μL of plasma. This score showed a moderate positive correlation with MS abundance (*ρ* = 0.45, *p* = 0.0080; Figure 3B). In the matched subset, HSPA8-scores were higher in stage I than in stages II and III CRC and were significantly higher in stage I CRC than in non-tumor participants (Figure 3C). Random forest classification using the score yielded internal cross-validation accuracies of 72.73% for stage I versus stages II–III CRC and 66.67% for stage I CRC versus non-tumor participants. The corresponding negative predictive values were 78.57% and 70.00% (Table S4). An exploratory reference cutoff of 0.035 was selected for each comparison by examining the observed scores alongside the random forest predictions (Figure 3D).

The ELISA cohort was expanded to 108 participants, comprising 16 with stage I CRC, 34 with stage II CRC, 40 with stage III CRC, four with stage IV CRC, and 14 non-tumor participants (Tables S2 and S3). HSPA8-scores were significantly higher in stage I than in stages II and IV CRC and in non-tumor participants. Scores were also higher in stage I than in stage III CRC, although this difference was not statistically significant (Figure 3E). In the expanded cohort, internal cross-validation accuracy was 74.47% for stage I versus stages II–IV CRC, with sensitivity of 25.00%, specificity of 84.62%, and a negative predictive value of 84.62%. For stage I CRC versus non-tumor participants, accuracy was 56.67%, sensitivity was 62.50%, specificity was 50.00%, and the negative predictive value was 53.85% (Figure 3F and Table S4). The exploratory reference cutoffs were 0.066 and 0.029, respectively. Neither HSPA8 concentration nor HSPA8-score showed a statistically significant association with age or sex in the expanded cohort (all *p* ≥ 0.411; Table S5). These results suggest the potential clinical translational significance of HSPA8 as a candidate plasma protein biomarker for early CRC.

### 2.4. Activating Histone Modification May Contribute to HSPA8 Upregulation in Early-Stage Patients with CRC

**Figure 4:**
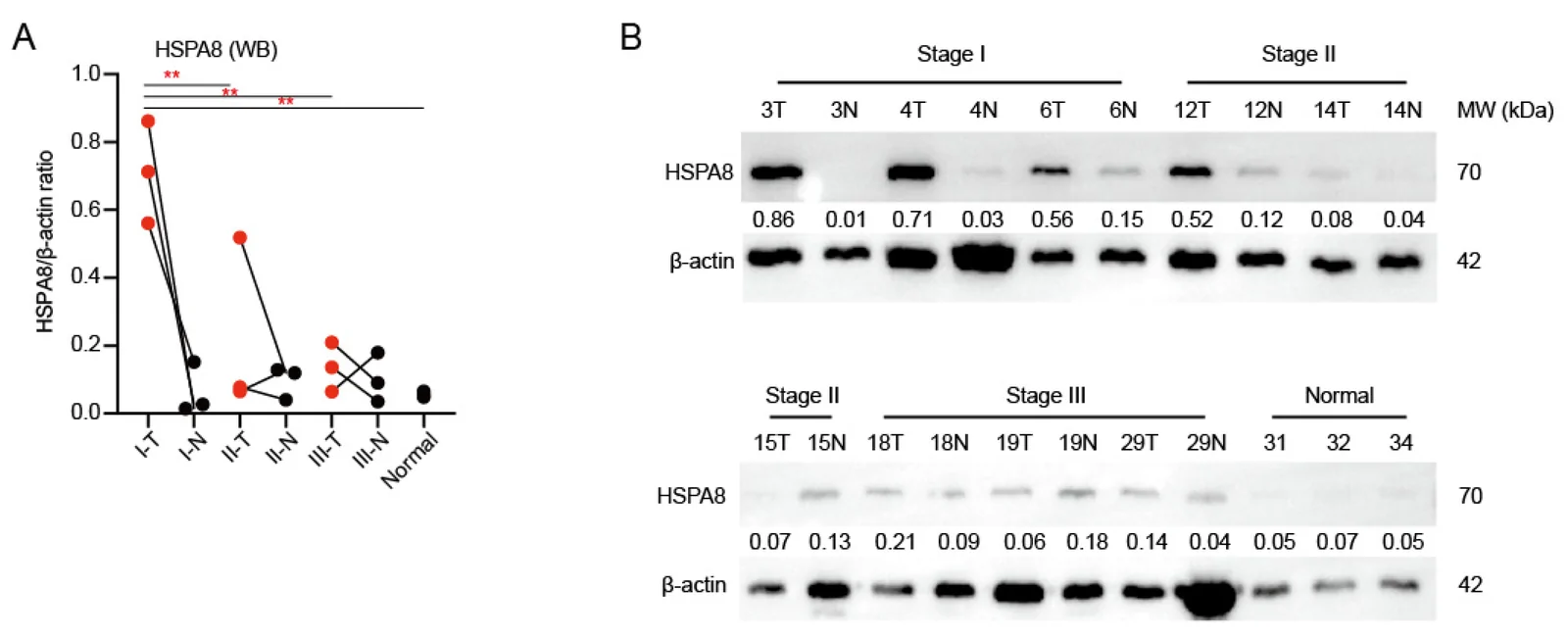
Western blot assessment of tissue HSPA8 abundance. (**A**) Boxplots summarizing HSPA8 abundance measured by Western blotting in CRC tumor tissues, paired distant-normal tissues, and intestinal tissues from non-tumor individuals (Lines connect paired samples from the same patient). ** *p* < 0.01 (**B**) Western blots showing HSPA8 and the β-actin loading control in the corresponding tissue groups.

**Figure 5:**
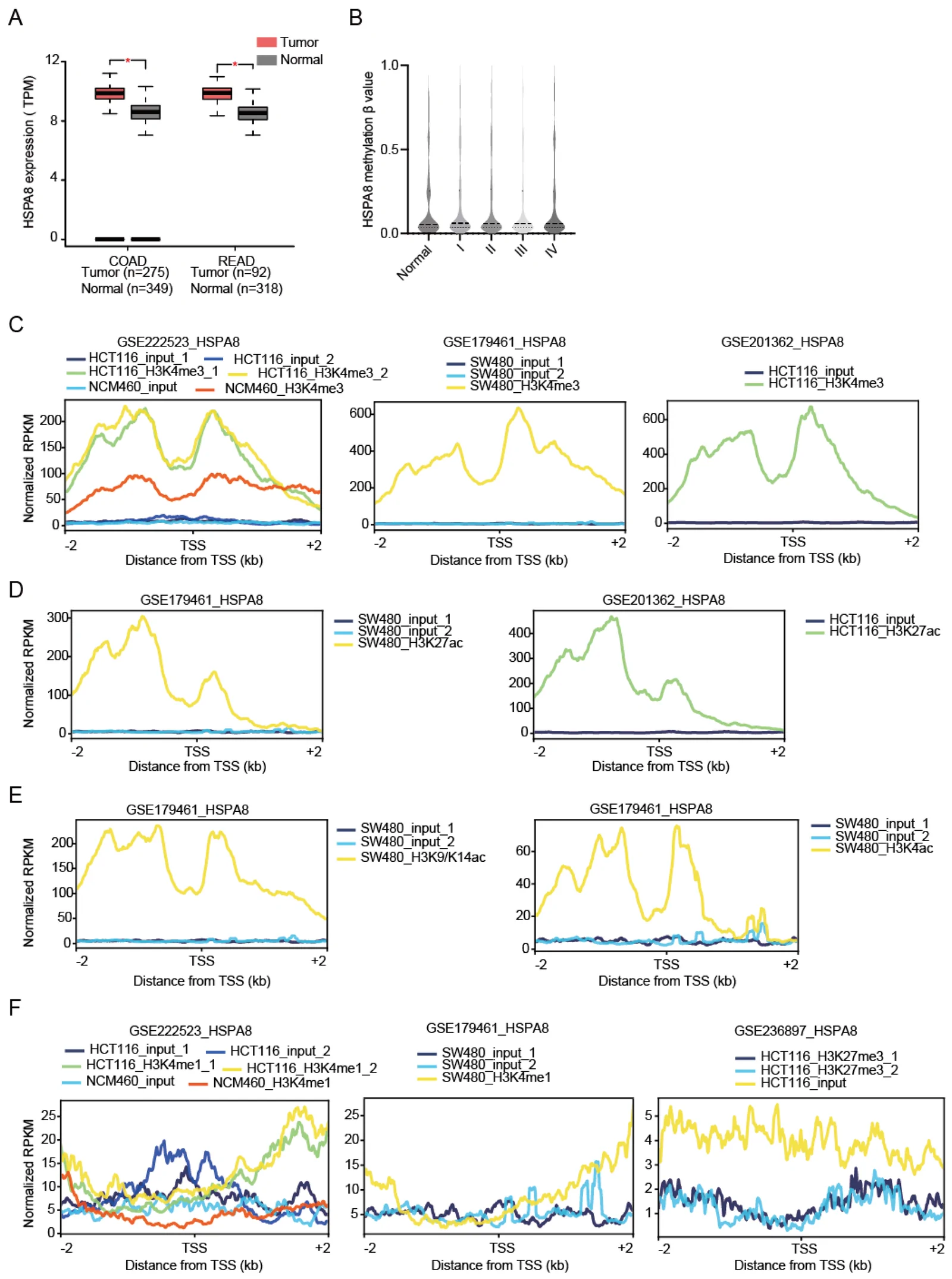
Mechanistic investigation of the rise of HSPA8 in early colorectal carcinoma. (**A**) Box plots demonstrate the expression of HSPA8 in CRC and normal tissues based on TCGA database (*, *p* < 0.05). (**B**) Boxplots demonstrating HSPA8 methylation in patients with different stages of colorectal carcinoma. (**C**) ChIP-seq signal profiles for H3K4me3 at the HSPA8 locus. (**D**) ChIP-seq signal profiles for H3K27ac at the HSPA8 locus. (**E**) ChIP-seq signal profiles for H3K4ac at the HSPA8 locus. (**F**) ChIP-seq signal profiles for H3K4me1 and H3K27me3 at the HSPA8 locus. (SW480: Colon Adenocarcinoma cell line; NCM460: normal colon mucosal epithelial cell line; HCT116: Colon Carcinoma).

Western blotting provides an antibody-based assessment of relative protein abundance, although quantitative interpretation depends on appropriate normalization and a suitable detection range [20]. Here, Western blotting was performed on CRC tumor tissues, paired distant-normal tissues, and intestinal tissues from three non-tumor individuals. HSPA8 abundance was significantly higher in stage I tumor tissues than in stage II and III tumor tissues and in the normal-tissue groups shown in Figure 4A,B. Because DNA methylation and chromatin alterations have been associated with changes in gene expression in CRC [21,22], we examined public expression and epigenomic datasets. HSPA8 transcript levels were significantly higher in colon and rectal adenocarcinoma tissues than in the corresponding normal-tissue reference groups (Figure 5A). The displayed methylation analysis showed no clear difference between normal tissues and tumors across clinical stages (Figure 5B). This observation does not exclude methylation-dependent regulation, particularly because HSPA8 locus methylation was not assessed in the tissue used for Western blotting.

Public ChIP-seq tracks showed greater H3K4me3 signal at the HSPA8 locus in HCT116 cells than in the nonmalignant colonic epithelial cell line NCM460, whereas H3K4me1 signal around the transcription start site was limited in both cell lines (Figure 5C,F). H3K4me3 enrichment was also observed in additional datasets from SW480 and HCT116 cells (Figure 5C). Other modifications associated with transcriptionally active chromatin, including H3K27ac, H3K9/K14ac, and H3K4ac, were present at the locus in CRC cell-line datasets that lacked nonmalignant colonic cell controls (Figure 5D,E). H3K27me3, a modification associated with transcriptional repression, showed weak signal at the locus in HCT116 cells (Figure 5F). These patterns are compatible with active chromatin at the HSPA8 locus and generate a hypothesis concerning its transcriptional regulation in CRC tissues may associated with activating histone modifications, especially H3K4me3.

### 2.5. Clinical Features Associated with HSPA8

Analysis of RNA-seq data of TCGA pan-cancer showed that HSPA8 was significantly overexpressed in breast invasive carcinoma (BRCA), colon adenocarcinoma (COAD), lymphoid neoplasm diffuse large B-cell lymphoma (DLBC), rectum adenocarcinoma (READ), skin cutaneous melanoma (SKCM), and thymoma (THYM) tumor tissues compared with paired normal tissues. Furthermore, survival analysis revealed that high HSPA8 expression predicted better survival in patients with CRC. We speculate that high HSPA8 expression in CRC may predict early-stage CRC with less malignancy (Figure S7).

## 3. Discussion

CRC is a common malignant tumor, and its proteomic profiles have been increasingly studied [23,24,25]. Zhang et al. identified five proteomic subtypes in the TCGA cohort. Two overlapped with the TCGA microsatellite instability/CpG island methylation phenotype transcriptomic subtype but showed distinct mutation, methylation, and protein expression patterns associated with different clinical outcomes. Comprehensive proteogenomic analyses provide a functional context for interpreting genomic alterations and offer a framework for understanding carcinoma biology [23]. In 2019, Vasaikar et al. compared the proteomes of paired tumors and adjacent normal tissues, generating a catalog of colon carcinoma-associated proteins, including known proteins and drug targets [24]. In 2020, Li et al. integrated genomic and proteomic analyses of 480 clinical tissue samples from 146 patients in a Chinese CRC cohort, including 70 patients with metastatic CRC. Their proteomic analysis distinguished three CRC subtypes with different clinical prognoses and molecular features [25]. However, tissue findings alone do not establish relevance to circulating biomarkers of early-stage CRC. Here, we combined stage-stratified plasma profiling with analyses of tumors and paired normal tissues to identify protein candidates preferentially elevated in stage I CRC relative to stages II and III CRC and non-tumor participants.

Our plasma findings overlapped with published CRC tissue proteomic datasets. The 98 genes corresponding to plasma proteins elevated in stage I CRC relative to non-tumor participants shared 44 members with Zhang’s proteomic subtype-signature union [23], one member (S100A11) with Vasaikar’s strictly selected 31-protein subset with higher tumor abundance [24], and 53 members with Li’s set of proteins differing between primary tumors and distant-normal tissues [25] (Figure S8A–C). HSPA8 was also elevated in the relevant Zhang subtype signatures and Li’s primary-tumor comparison, consistent with its increased abundance in our stage I plasma samples. Together with the tissue findings in our cohort, this concordance supports an association between HSPA8 and CRC and provides a rationale for further assessing its potential as a circulating biomarker for early-stage disease. These published datasets were derived from CRC tissues, whereas our discovery candidates were identified in plasma. Differences in biological compartments, clinical comparisons, and selection criteria may therefore contribute to discrepancies in protein abundance patterns.

Liquid biopsy has attracted increasing attention in solid tumor research [26,27,28,29]. This approach involves the analysis of carcinoma-related molecules that can be readily obtained from a patient’s blood sample. Proteomic studies of body fluids provide a relevant comparison for our plasma findings. Sun et al. profiled 657 urine samples and 993 tissue samples from healthy controls and patients with CRC at different metastatic risks. Combining their diagnostic signature with FIT increased sensitivity by 21.2 percentage points in the training set and 43.7 percentage points in the validation set compared with FIT alone [30]. Their final three-protein diagnostic panel (CORO1C, ARPC5, and RAD23B) did not overlap with our seven plasma candidates. However, the mapped portions of their differential protein lists for nonmetastatic, lymph-node-metastatic, and distant-metastatic CRC shared 10, 12, and 14 genes, respectively, with the 98 genes corresponding to plasma proteins elevated in stage I CRC in our study (Figure S8D–F). Nonmetastatic CRC is not equivalent to stage I disease, and urine and plasma represent different biological compartments. The overlap therefore suggests partially shared protein alterations across body fluids, without establishing a common source or concordant regulation.

In this study, we performed high-throughput proteomic profiling of tumors and paired normal tissues, together with matched plasma samples from patients with stages I–III CRC. Thousands of proteins were detected in tissues and hundreds in plasma. Integrated analysis of the tissue and plasma proteomic data suggested that some differentially abundant plasma proteins may be associated with tumor tissue, as illustrated by the three candidates examined further. Seven proteins had higher plasma abundance in stage I CRC than in stages II and III CRC and non-tumor participants. Tissue analysis identified three of these proteins (HSPA8, ALDOA, and PDLIM1) with higher abundance in stage I CRC tumors, suggesting an association with early-stage CRC. In the discovery comparison, machine learning classification using HSPA8 alone achieved the same accuracy as the three-protein model. These findings supported prioritizing HSPA8 as a potential plasma protein marker for early CRC.

Analysis of TCGA data revealed significantly higher HSPA8 expression in colorectal tumors than in normal tissues and significantly better overall survival among patients with CRC with high HSPA8 expression (*p* < 0.05). The molecular chaperone HSPA8 contributes to protein homeostasis through protein folding and chaperone-mediated autophagy [31,32]. HSPA8 has also been studied in triple-negative breast, liver, and bladder cancers [33,34,35]. In a breast cancer model, HSPA8 overexpression reduced PD-L1 expression, inhibited tumor growth, and promoted antitumor immunity [36]. These findings support the biological relevance of HSPA8. Our analyses of public expression and chromatin datasets generated a further hypothesis concerning HSPA8 regulation. The observed expression patterns and activating histone modifications, particularly H3K4me3 enrichment, are compatible with a transcriptional contribution to HSPA8 upregulation.

Proteomic profiling requires complex sample preparation and advanced instrumentation, which limit its clinical translation. We therefore first assessed HSPA8 by ELISA in 32 participants with proteomic measurements. HSPA8 concentration showed a weak, non-significant association with MS abundance. Normalizing the concentration to the total protein amount in 10 μL of plasma to derive the HSPA8-score yielded a stronger, statistically significant association. HSPA8-scores were significantly higher in stage I CRC than in non-tumor participants. In the expanded cohort of 108 participants, scores were significantly higher in stage I than in stages II and IV CRC and in non-tumor participants.

Our study focused on stage I CRC. We selected plasma candidates elevated relative to stages II and III CRC and non-tumor participants, followed by tissue and ELISA assessment. Fung et al. reported a stage I sensitivity of 52% for serum PKM2 alone and 59% for a DKK3/IGFBP2/PKM2 panel at 95% specificity in their test cohort [37]. Bhardwaj et al. developed a 12-protein plasma signature for early CRC, defined as stages I and II combined, with a bootstrap-adjusted discovery AUC of 0.91 and an independently validated AUC of 0.75 [15]. By examining stage I as a distinct group, our focus on preferential stage I elevation and direct classification against later-stage CRC complements these detection approaches.

This study identified HSPA8 as a candidate plasma protein biomarker preferentially elevated in stage I CRC, supported by tissue protein measurements and subsequent ELISA assessment. In the discovery comparison of stage I CRC with non-tumor participants, HSPA8 alone achieved 88.9% classification accuracy, matching the three-protein model. In the expanded ELISA cohort, internally cross-validated random forest classification using the HSPA8-score yielded a sensitivity of 62.50% and specificity of 50.00% for stage I CRC versus non-tumor participants, and a sensitivity of 25.00% and specificity of 84.62% for stage I versus stages II–IV CRC. These findings support further investigation of HSPA8 for stage I detection, although specificity against non-tumor participants and sensitivity against later-stage CRC require improvement. Given the exploratory candidate selection and internal evaluation, validation in larger, independent multicenter cohorts using a standardized assay and prespecified thresholds is needed to establish clinical value.

## 4. Materials and Methods

### 4.1. Clinical Sample Collection

A clinical sample bank was established with written informed consent from patients undergoing CRC surgery at the Department of Colorectal Surgery, Tianjin Union Medical Center, Tianjin, China. Chinese patients provided primary tumor specimens, matched normal tissues collected 5 cm from the tumor margin (*n*), and preoperative blood samples. Within 30 min of resection, tissue specimens were placed in sterile cryovials, snap-frozen in liquid nitrogen, and stored at −80 °C. Plasma and blood cells were also stored at −80 °C until analysis. Plasma proteomics included 24 patients with CRC and 10 non-tumor participants; tissue proteomics included 24 paired tumor and distant-normal specimens and four non-tumor intestinal specimens. The ELISA cohort comprised 108 participants, including 94 patients with CRC and 14 non-tumor participants. The study was conducted in accordance with the Declaration of Helsinki, and the protocol was approved by the Ethics Committee of Tianjin Union Medical Center (No. 2024-B112; Tianjin, China) on 16 September 2024.

### 4.2. Protein Extraction and Digestion

Tissue proteins were extracted from 20–50 mg of wet tissue in SDT buffer (Coolaber Science & Technology, Cat#SL1029, Beijing, China). Samples were heated at 95 °C for 5 min and sonicated for 3 min using alternating 5 s pulses and 10 s pauses. After centrifugation at 14,000× *g* for 10 min, the soluble fraction was transferred to fresh tubes. Protein concentration was measured using the Bradford assay (Thermo Fisher, Cat#23200, RRID:SCR_008452, Waltham, MA, USA). Plasma albumin was depleted using the Albumin Depletion Kit (Thermo Fisher, Cat#85160, RRID:SCR_008452) to reduce interference with MS detection. Plasma protein concentration was measured using the BCA assay (Thermo Fisher, Cat#23227, RRID:SCR_008452) before depletion.

To recover unbound proteins, the column was transferred to a new tube and washed with 50 µL of Binding/Wash Buffer per 200 µL of resin. Protein amounts were then determined using the BCA assay. A 40 μg aliquot from each tissue or plasma sample was processed by filter-aided sample preparation (FASP) [38] using the FASP Protein Digestion Kit (Abcam, Cat#ab270519, RRID:SCR_012931, Cambridge, UK). Peptide mixtures were recovered by centrifugation and dried in a Speed Vac.

### 4.3. Proteomic Measurements and Analysis

Peptides v2.4.6 were separated using a Thermo Scientific EASY-nLC™ 1200 nano LC system (Thermo Fisher) operated in trap-elute mode. Eluting peptides were analyzed using a Thermo Scientific Orbitrap Eclipse mass spectrometer equipped with FAIMS Pro (both Thermo Fisher). Repeated plasma sampling was not performed, and patient plasma samples were not pooled.

The mobile phases were aqueous 0.1% formic acid (A) and 0.1% formic acid in 80% acetonitrile (B). Peptides were loaded onto a Thermo Scientific Acclaim PepMap 100 C18 trap column (75 μm × 2 cm, 3 μm, 100 Å) at 10 μL/min for 3 min and separated on a Thermo Scientific Acclaim PepMap RSLC analytical column (75 μm × 25 cm, 1.9 μm, 100 Å) at 550 nL/min. The proportion of B increased from 8% to 30% over 127 min and then from 30% to 90% over 16 min, followed by a 14 min wash.

Positive-ion full scans covered *m*/*z* 350–2000 at a resolution of 60,000 at *m*/*z* 200. The full-scan automatic gain control (AGC) target was 4e5, and the maximum injection time was 50 ms. Data-dependent MS2 acquisition used a top 1 s setting, with previously selected precursors excluded for 30 s. FAIMS Pro data were acquired at −45 V and −65 V. EThcD acquisition combined calibrated, charge-dependent ETD settings with supplemental HCD activation at 27% normalized collision energy (NCE). The EThcD resolution was 30,000 at *m*/*z* 200, with an AGC target of 4e5 and a maximum injection time of 400.

Raw MS data were processed using Proteome Discoverer v2.4 (Thermo Fisher Scientific, RRID:SCR_008452, Waltham, MA, USA) and searched against reviewed human entries in the UniProt protein sequence database (202104) (Version 2021_04) [Released 29 September 2021]. The initial search also included a database of common contaminants. Searches specified trypsin digestion and allowed two missed cleavages. The proteomic data are available in the PRIDE repository (RRID:SCR_003411), a partner of the ProteoXchange Consortium, under accession PXD055961. Both the raw mass spectrometry files (.raw) and the PD output files were uploaded to this dataset.

### 4.4. Differentially Abundant Proteins and Enrichment Analysis

Protein identifications from Proteome Discoverer were filtered in two steps: entries flagged as Contaminant = True were removed, followed by proteins supported by fewer than two unique peptides. Plasma protein abundances were compared across clinical-stage groups to identify candidates elevated in stage I CRC. Proteins were considered elevated in stage I CRC at a Wilcoxon test *p*-value < 0.05 and a fold change > 1.5. Pathway enrichment was performed using Metascape v3.5.20260701 (https://metascape.org/gp/index.html#/main/step1, accessed on 22 October 2026, RRID:SCR_016620). Input sets contained only proteins significantly elevated in the respective comparisons (30, 60, and 98 proteins); downregulated proteins were excluded. Protein-set overlaps were visualized using Ghent University’s online Venn diagram tool (https://bioinfogp.cnb.csic.es/tools/venny/, accessed on 22 October 2026).

### 4.5. Enzyme-Linked Immunosorbent Assay (ELISA)

Human 70kDa Heat Shock Protein 8 (HSPA8) ELISA kit (ELK Biotechnology, Cat#ELK1309, Sugar Land, TX, USA) was usde to marsure plasma HSPA8 in participants with different stages of CRC and non-tumor participants. Briefly, plasma samples were diluted 10-fold before analysis. Samples and kit reagents were brought to room temperature. Each well received 100 μL of standard working buffer or plasma and was incubated at 37 °C for 80 min. Wells were emptied, washed three times with 200 μL of Wash Buffer per well, and patted dry. Biotinylated Antibody Buffer (100 μL) was then added to each well, followed by incubation at 37 °C for 50 min. The liquid was removed, and wells were washed three times with 200 μL of Wash Buffer per well and patted dry. HRP enzyme working solution (100 μL) was added to each well, followed by incubation at 37 °C for 50 min.

After HRP incubation, wells were emptied, washed five times with 200 μL of Wash Buffer per well, and patted dry. TMB (90 μL) was added to each well for color development at 37 °C for 20 min. The reaction was stopped by adding 50 μL of termination buffer per well, and absorbance was recorded immediately at 450 nm. Cruve Export Pro v2.7.3 was used to construct the standard curve. The HSPA8-score was calculated by dividing the dilution-corrected HSPA8 concentration by the total protein amount in 10 μL of plasma.

### 4.6. Correlations of Plasma Protein Abundance and ELISA Measurements with Age and Sex

Associations of plasma protein measurements with age and sex were assessed using Spearman’s rank correlation. The proteomic analysis included ALDOA, HSPA8, and PDLIM1 abundances from 34 plasma samples. The ELISA analysis included HSPA8 concentrations (ng/mL) and HSPA8-scores from 108 participants. Age was treated as a continuous variable, and sex was coded as female = 0 and male = 1. Tied observations were assigned average ranks. Spearman’s correlation coefficients (ρ) and two-sided asymptotic *p*-values were calculated using scipy.stats.spearmanr. Multiple testing was controlled using the Benjamini–Hochberg procedure, applied separately to the six proteomic comparisons and four ELISA comparisons. Adjusted *p*-values were reported as q_BH, with q_BH < 0.05 considered statistically significant.

### 4.7. Random Forest Classification and Exploratory Score Cutoff

Random forest classification was performed in Weka v3.8.6 (RRID:SCR_001214) [39] to prioritize biomarkers and subsequently evaluate the HSPA8-score. Following candidate assessment in plasma and paired tissues, ALDOA, HSPA8, and PDLIM1 were evaluated individually and in combination using their plasma MS abundances to distinguish stage I CRC (*n* = 8) from non-tumor participants (*n* = 10). Input data comprised the abundances of individual proteins or protein combinations in these plasma samples. ELISA assessment was first performed in 32 participants with matched plasma proteomic and ELISA measurements and then extended to 108 participants, including the initial group. The HSPA8-score was defined as the dilution-corrected HSPA8 concentration divided by the total protein amount in 10 μL of plasma. Matched-subset comparisons were stage I CRC (*n* = 8) versus stages II–III CRC (*n* = 14), and stage I CRC (*n* = 8) versus non-tumor participants (*n* = 10). Expanded-cohort comparisons were stage I CRC (*n* = 16) versus stages II–IV CRC (*n* = 78: 34 stage II, 40 stage III, and 4 stage IV), and stage I CRC (*n* = 16) versus non-tumor participants (*n* = 14). Each comparison was evaluated separately, with stage I CRC treated as the positive class.

The classifier was run using “weka.classifiers.trees.RandomForest -P 100 -I 100 -num-slots 1 -K 0 -M 1.0 -V 0.001 -S 1”. The forest contained 100 trees, with bootstrap bags equal in size to the corresponding training set, one execution slot, a minimum total instance weight per leaf of 1.0, and classifier seed 1. Setting K = 0 invoked automatic feature-subset selection, with nominal K = floor(log2(*p*)) + 1 for p predictors; the single-predictor score models used only the HSPA8-score. The V = 0.001 option specifies a variance criterion for numerical-class tree splitting.

Each comparison was evaluated using stratified 10-fold cross-validation. The model was fitted on nine folds and used to predict the remaining fold, with the process repeated across all ten folds. Performance was calculated from pooled out-of-fold predictions. The cohorts were not divided into fixed, separate training, validation, and test sets. Because the matched subset was included in the expanded cohort, both assessments provided internal evaluation.

To select exploratory score cutoffs, participants in each comparison were ordered by HSPA8-score, and the ordered scores were examined alongside random forest classification results. Reference cutoffs were selected manually between adjacent observed scores and reported to three decimal places. The matched-subset cutoffs were 0.035 for both comparisons. The expanded-cohort cutoffs were 0.066 for stage I versus stages II–IV CRC and 0.029 for stage I CRC versus non-tumor participants.

### 4.8. GEPIA Analysis of HSPA8 Expression and Prognosis in CRC

HSPA8 expression and its association with prognosis in CRC were examined using GEPIA (http://gepia2.cancer-pku.cn/#analysis, accessed on 22 October 2026, RRID:SCR_018294) and tumor and normal-tissue transcriptome data from TCGA. We compared HSPA8 expression between tumor and normal tissues and assessed its association with overall survival (OS). For survival analysis, participants were divided into two groups at the median expression value. Hazard ratios (HRs), 95% confidence intervals (CIs), and chi-square test *p*-values were calculated. Statistical significance was defined as *p* < 0.05.

### 4.9. Western Blot

Protein extracts were separated by 10% Acr-Bis SDS-PAGE and transferred to polyvinylidene difluoride (PVDF) membranes (Millipore, Cat#ISEQ00005, RRID:SCR_008983, Burlington, MA, USA). Membranes were blocked in 5% nonfat milk at room temperature for 2 h and incubated with primary antibodies at 4 °C overnight. The primary antibodies were rabbit anti-HSPA8 (1:2000; ELK, es2569) and mouse anti-β-Actin (1:5000; ABclonal, Cat#AC004, RRID:AB_2737399, Woburn, MA, USA). The following day, membranes were incubated at room temperature for 1 h with goat anti-rabbit IgG (H+L) (ABclonal, Cat#AS014, RRID:AB_2769854) or goat anti-mouse IgG (H+L) (ABclonal, Cat#AS003, RRID:AB_2769851), each diluted 1:5000. Bands were detected using Supersignal West Pico Chemiluminescent Substrate (Millipore, Cat#WBKLS0500, RRID:SCR_008983).

### 4.10. ChIP-Seq Analysis

Public ChIP-seq data were obtained from GSE236897 [40], GSE222523 [41], GSE201362 [42], and GSE179461 [42]. SRA files were converted to FASTQ format, and low-quality sequence data were trimmed before alignment. Cleaned reads were aligned to the human GRCh38 assembly using Bowtie2 (RRID:SCR_016368) [43] with the parameters “--local -k 1”. The -k 1 option limited the number of reported valid alignments to one per read. BAM files were generated using SAMtools v1.7 (RRID:SCR_002105). Scatterplots, correlation plots, and heatmaps were generated using deepTools (RRID:SCR_016366) [44].

## 5. Conclusions

We collected tumors and paired normal tissues from patients with stages I–III CRC, together with matched plasma samples and plasma from non-tumor participants. We combined high-throughput proteomic profiling with comparisons of tumor and normal-tissue protein abundance within each patient, identification of differentially abundant proteins in stage I plasma, and machine learning classification. Seven plasma proteins were elevated in stage I CRC relative to stages II and III CRC and non-tumor participants; three of these proteins (HSPA8, ALDOA, and PDLIM1) were also elevated in stage I tumors. HSPA8 alone achieved the same classification accuracy as the three-protein model in the discovery comparison. Public ChIP-Seq data implied that activating histone modifications may contribute to HSPA8 upregulation. To assess its potential for clinical translation, we measured HSPA8 by ELISA and normalized its concentration to the total protein amount in 10 μL of plasma to derive the HSPA8-score. Scores were significantly higher in stage I than in stages II and IV CRC and in non-tumor participants. Scores were also higher in stage I than in stage III CRC, although this difference was not statistically significant. In the expanded cohort of 108 participants, internally cross-validated random forest classification using the HSPA8-score achieved accuracies of 74.47% for stage I versus stages II–IV CRC and 56.67% for stage I CRC versus non-tumor participants. The corresponding exploratory reference cutoffs were 0.066 and 0.029. Sensitivity and specificity were 25.00% and 84.62% against later-stage CRC, and 62.50% and 50.00% against non-tumor participants. These findings identify HSPA8 as a candidate marker for early CRC detection. Validation in larger, independent multicenter cohorts using standardized assays and prespecified thresholds is needed before clinical application.

## Data Availability

The mass spectrometry proteomic data have been deposited in the ProteoXchange Consortium via the PRIDE partner repository (RRID:SCR_003411) under dataset identifier PXD055961. The public ChIP-seq datasets GSE236897, GSE222523, GSE201362, and GSE179461 were used in this study. Other data are available from the corresponding author upon reasonable request.

## Supplementary Materials

The following supporting information can be downloaded at: https://www.mdpi.com/article/10.3390/ijms27219630/s1.
