# Supplementary material for: Identification of Novel Plasma Protein Biomarkers for Early Clinical Stage Colorectal Carcinoma by High-Throughput Proteomics
Source: Int J Mol Sci. 2026 Oct 28;27(21):9630. doi: 10.3390/ijms27219630 (PMC13762703; doi:10.3390/ijms27219630)
Supplement: Supplementary file 1 [file ijms-27-09630-s001.zip › ijms-4611922-supplementary figure.pdf]

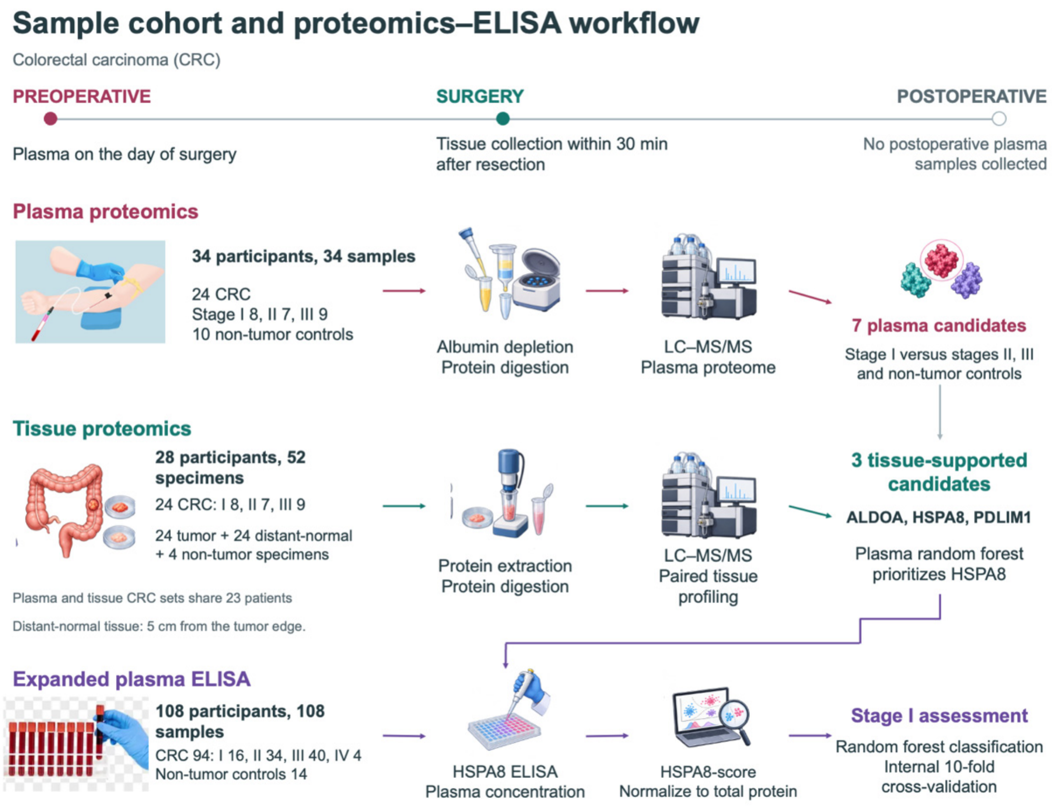

Figure S1. Study cohorts and proteomic–ELISA workflow. Plasma proteomics included 24 patients with colorectal carcinoma (CRC) and 10 non-tumor controls. Tissue proteomics included 24 paired tumor and distant-normal tissue samples and four non-tumor specimens; the plasma and tissue CRC cohorts shared 23 patients. The seven plasma candidates were narrowed to three proteins supported by tissue findings (ALDOA, HSPA8, and PDLIM1), and random forest analysis prioritized HSPA8. HSPA8 was subsequently measured by enzyme-linked immunosorbent assay (ELISA) in 94 patients with CRC and 14 controls. The HSPA8-score, normalized to total plasma protein, was evaluated for stage I classification using internal 10-fold cross-validation.

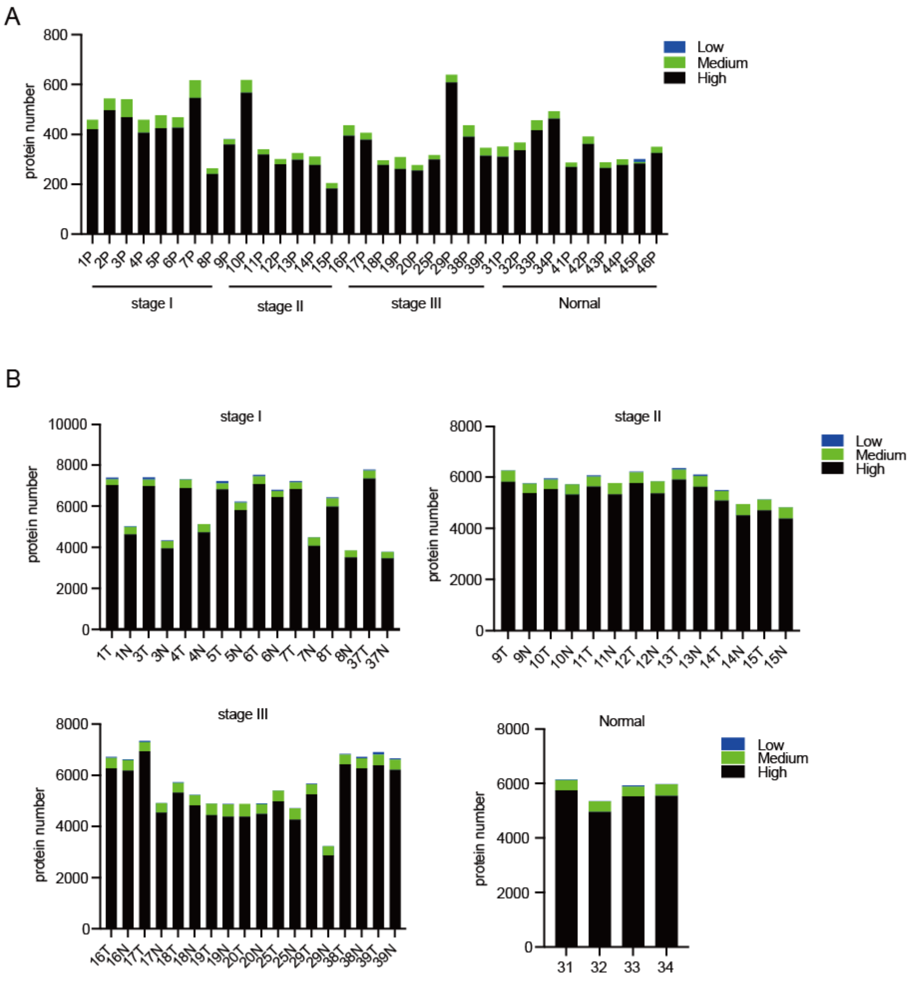

Figure S2. Protein identification counts and confidence levels in plasma and tissue samples.A. Numbers of proteins identified in individual plasma samples from patients with stage I, II, or III CRC and non-tumor controls.**B.** Numbers of proteins identified in tumors (T) and paired distant-normal tissues (N) from patients with stage I, II, or III CRC and in intestinal tissues from non-tumor controls.Each stacked bar represents one sample. Blue, green, and black indicate low-, medium-, and high-confidence protein identifications, respectively, as assigned by Proteome Discoverer v2.4.

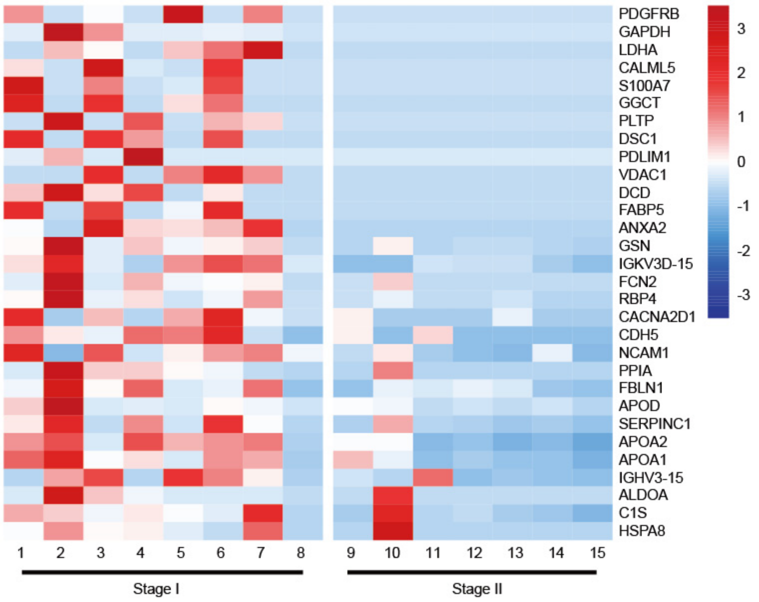

Figure S3. Plasma proteins elevated in stage I relative to stage II CRC. The heatmap shows proteins with significantly higher plasma abundance in patients with stage I CRC (n = 8) than in those with stage II CRC (n = 7). Red and blue indicate high and low protein abundance, respectively. Selection criteria were fold change > 1.5 and $P < 0.05$.

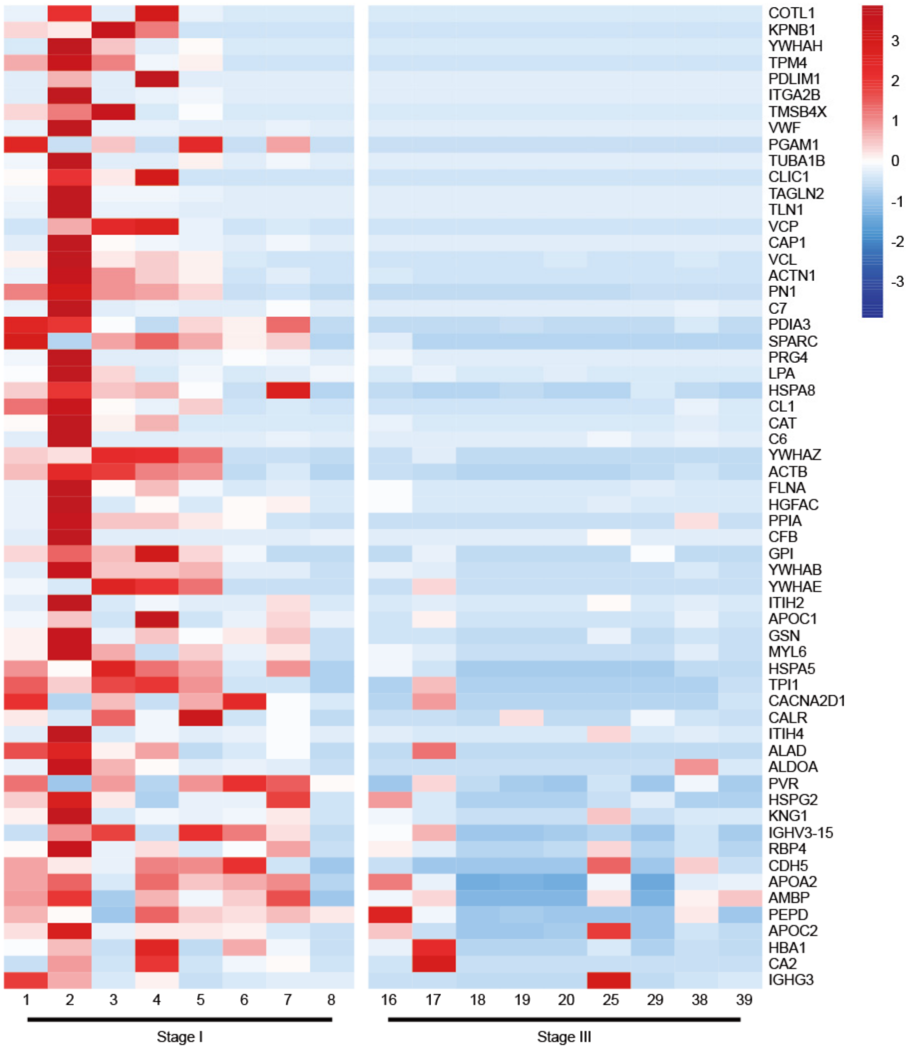

Figure S4. Plasma proteins elevated in stage I relative to stage III CRC. The heatmap shows proteins with significantly higher plasma abundance in patients with stage I CRC (n = 8) than in those with stage III CRC (n = 9). Red and blue indicate high and low protein abundance, respectively. Selection criteria were fold change > 1.5 and P < 0.05.

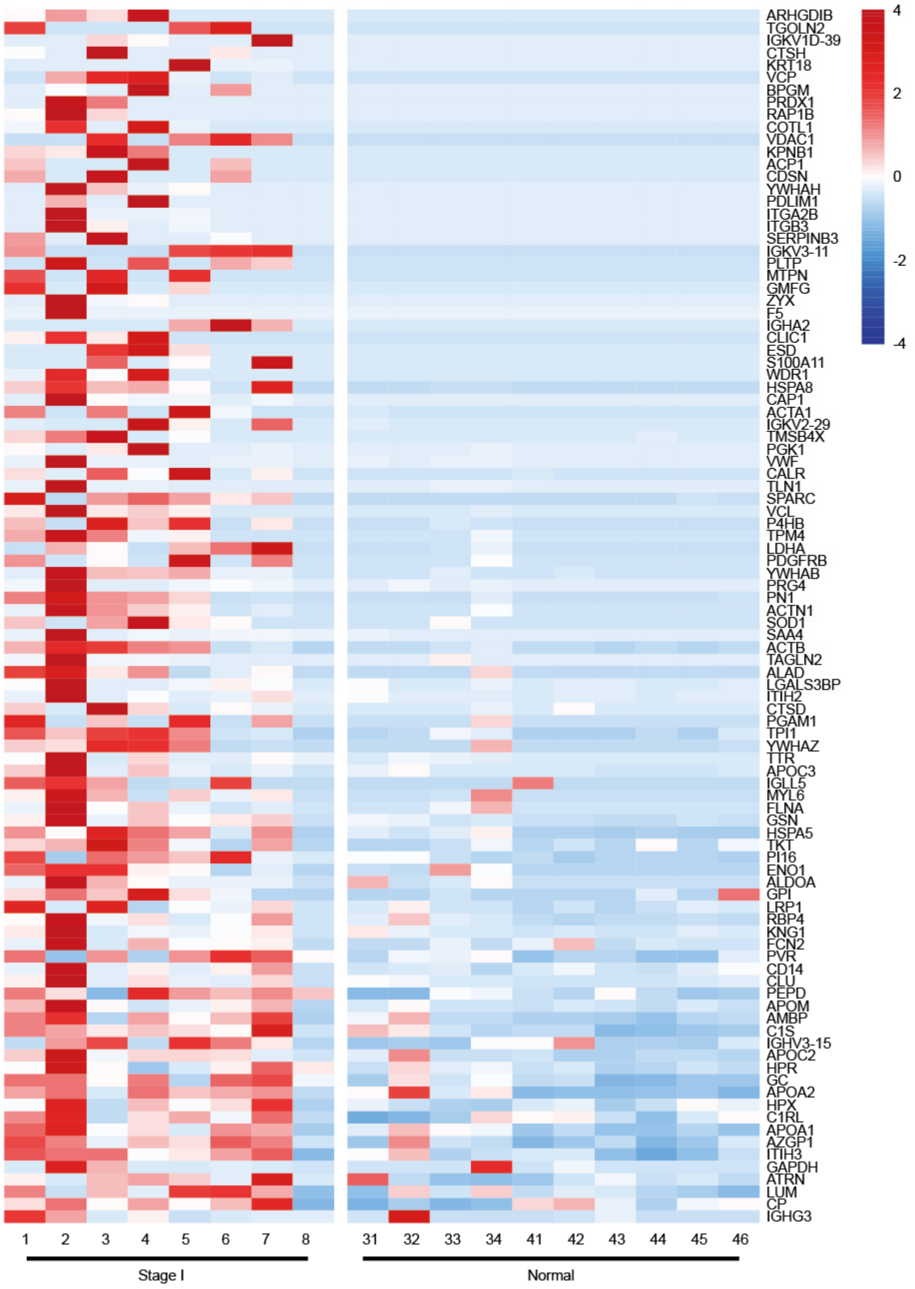

Figure S5. Plasma proteins elevated in stage I CRC relative to non-tumor participants. The heatmap shows proteins with significantly higher plasma abundance in patients with stage I CRC (n = 8) than in non-tumor participants (n = 10). Red and blue indicate high and low protein abundance, respectively. Selection criteria were fold change > 1.5 and P < 0.05.

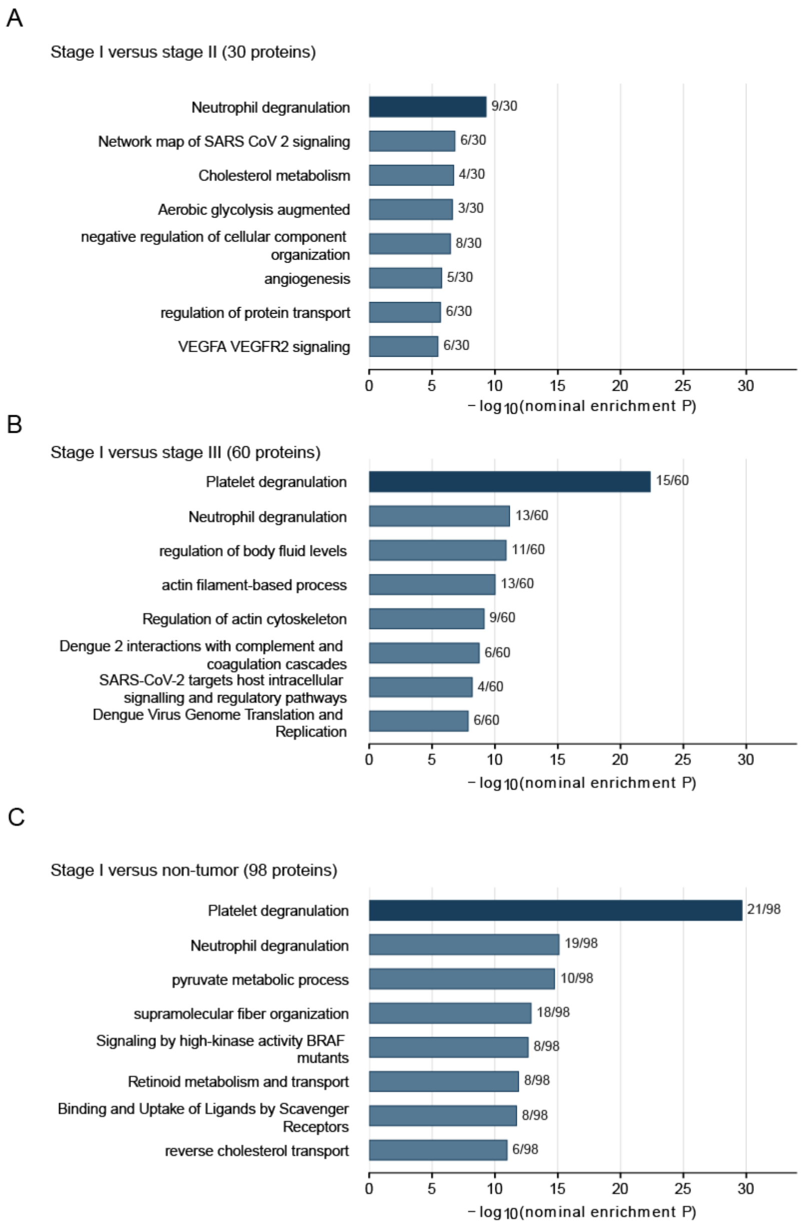

Figure S6. Pathway enrichment analysis of plasma proteins significantly elevated in stage I CRC.(A–C) Pathway enrichment analyses of plasma proteins with significantly higher abundance in patients with stage I CRC than in patients with stage II CRC **(A)**, patients with stage III CRC **(B)**, or non-tumor controls (C). Each analysis included only proteins significantly elevated in stage I CRC in the corresponding comparison.

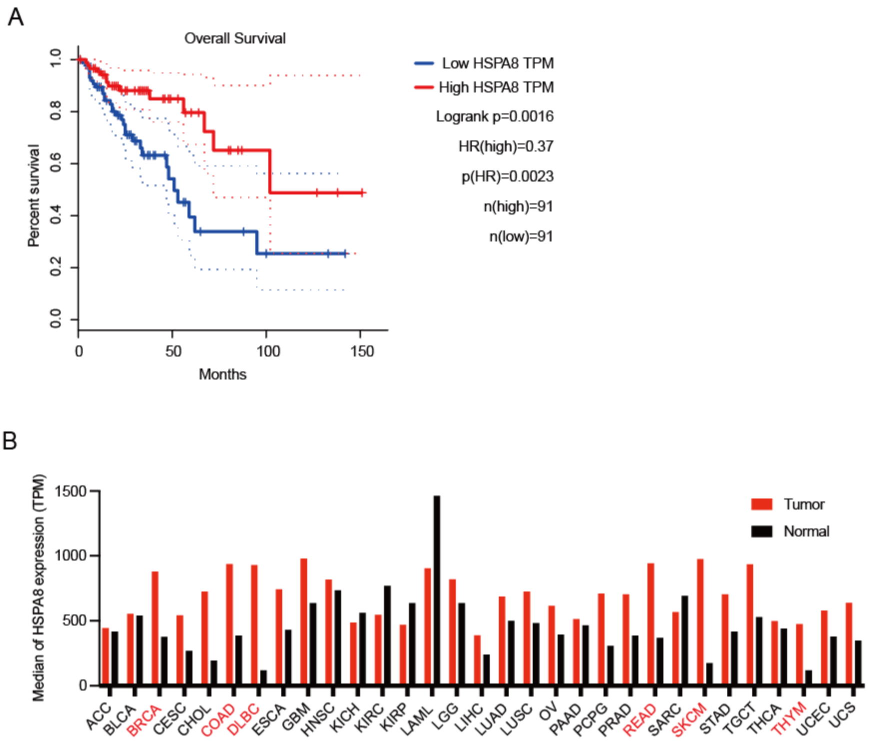

Figure S7. Clinical associations of HSPA8.A. Association of HSPA8 expression with survival in patients with CRC.**B**. Bar plots showing HSPA8 expression in tumor and normal tissues across cancer types using TCGA data. Red labels indicate cancer types with statistically significant differences.Abbreviations: ACC, adrenocortical carcinoma; BLCA, bladder urothelial carcinoma; BRCA, breast invasive carcinoma; CESC, cervical squamous cell carcinoma and endocervical adenocarcinoma; CHOL, cholangiocarcinoma; COAD, colon adenocarcinoma; DLBC, lymphoid neoplasm diffuse large B-cell lymphoma; ESCA, esophageal carcinoma; GBM, glioblastoma multiforme; HNSC, head and neck squamous cell carcinoma; KICH, kidney chromophobe; KIRC, kidney renal clear cell carcinoma; KIRP, kidney renal papillary cell carcinoma; LAML, acute myeloid leukemia; LGG, brain lower grade glioma; LIHC, liver hepatocellular carcinoma; LUAD, lung adenocarcinoma; LUSC, lung squamous cell carcinoma; OV, ovarian serous cystadenocarcinoma; PAAD, pancreatic adenocarcinoma; PCPG, pheochromocytoma and paraganglioma; PRAD, prostate adenocarcinoma; READ, rectum adenocarcinoma; SARC, sarcoma; SKCM, skin cutaneous melanoma; STAD, stomach adenocarcinoma; TGCT, testicular germ cell tumors; THCA, thyroid carcinoma; THYM, thymoma; UCEC, uterine corpus endometrial carcinoma; UCS, uterine carcinosarcoma.

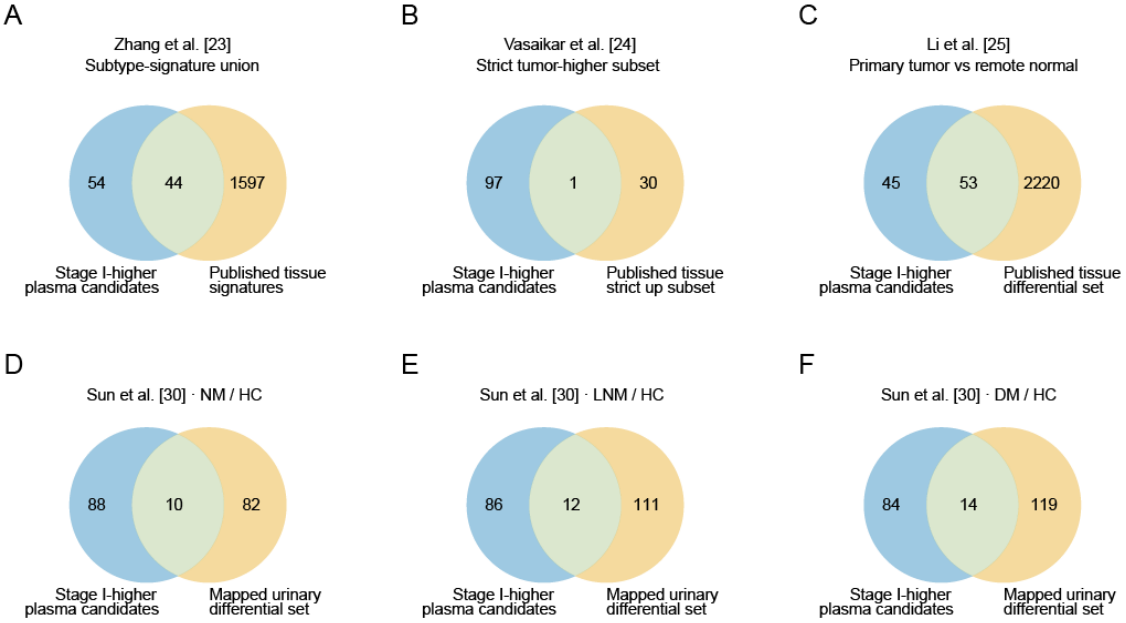

Figure S8. Overlap of stage I CRC plasma candidates with published tissue and urinary proteomic datasets. Venn diagrams compare the 98 gene symbols corresponding to plasma proteins with nominally elevated abundance in stage I CRC relative to non-tumor participants with published protein sets. Blue circles represent our plasma candidates, and yellow circles represent the published sets.(A–C) Comparisons with CRC tissue proteomic sets from Zhang et al. [23], Vasaikar et al. [24], and Li et al. [25], respectively. The proteomic subtype-signature union, strictly selected subset with higher tumor abundance, and set of proteins differing between primary tumors and distant-normal tissues shared 44, one (S100A11), and 53 genes, respectively, with our plasma candidates. Panel B includes only the selected 31-protein subset.(D–F) Comparisons with mapped urinary differential protein sets from Sun et al. [30] for nonmetastatic CRC (NM), lymph-node-metastatic CRC (LNM), and distant-metastatic CRC (DM) versus healthy controls (HC), respectively. These comparisons identified 10, 12, and 14 shared genes, respectively.
