# Supplementary material for: Identification of Novel Plasma Protein Biomarkers for Early Clinical Stage Colorectal Carcinoma by High-Throughput Proteomics
Source: Int J Mol Sci. 2026 Oct 28;27(21):9630. doi: 10.3390/ijms27219630 (PMC13762703; doi:10.3390/ijms27219630)
Supplement: Supplementary file 1 [file ijms-27-09630-s001.zip › ijms-4611922-supplementary table.pdf]

Supplementary Tables

Table S1. Patient clinical information

Elisa.No	Sex	Age	Stage	ptx
1	male	**57**	I	pt1nomx
2	male	**49**	I	pt2n0mx
3	female	**62**	I	pt2n0mx
4	female	**73**	II	pt4nomx
5	female	**90**	II	pt3n0mx
6	male	**77**	III	pt3n1mx
7	female	**70**	III	pt4n1mx
8	female	**48**	non	non
9	female	**53**	non	non
10	male	**58**	I	pt1n0mx
11	female	**34**	non	non
12	female	**62**	non	non
13	male	**63**	III	pt3n1mx
14	male	**65**	II	pt4n0mx
15	male	**66**	I	pt2n0mx
16	female	**65**	III	pt3n1mx
17	female	**58**	III	t4n2mx
18	female	**76**	II	t4n0mx
19	male	**52**	I	pt1n0mx
20	male	**47**	III	pt3n1mx
21	male	**62**	II	pt3n0mx
22	male	**47**	III	pt4n2mx
23	female	**58**	IV	pt3n0m1
24	male	**41**	II	t3n0mx
25	female	**47**	III	pt4n2mx
26	male	**55**	III	t2n1mx
27	male	**66**	III	t3n1mx
28	male	**57**	I	t1n0mx
30	male	**67**	I	t2n0mx

31	female	**71**	II	pt3n0mx
32	female	**76**	II	pt3n0mx
33	male	**69**	III	pt3n1mx
34	male	**57**	III	t4n1mx
35	female	**71**	III	pt3n2mx
36	male	**54**	III	pt2n2amx
37	female	**60**	I	pt1n0mx
38	female	**65**	IV	pt3n1m1
39	male	**46**	III	pt4n1mx
40	male	**65**	III	pt4n1mx
41	male	**70**	IV	t4n1m1
42	male	**65**	III	t3n1mx
43	male	**48**	I	pt2n0mx
44	female	**52**	III	pt4n1mx
45	male	**73**	II	pt3n0mx
46	male	**74**	II	pt4n0mx
47	male	**56**	II	t4n0mx
49	male	**45**	II	pt3n0mx
50	female	**53**	II	pt3n0mx
51	female	**85**	II	pt3n0mx
52	female	**71**	II	pt4bn0mx
53	female	**66**	III	t4n2mx
54	male	**65**	III	pt4n1mx
55	female	**63**	III	t3n1mx
56	female	**75**	II	pt4an0mx
57	female	**64**	III	pt3n1mx
58	male	**44**	II	pt3n0mx
59	female	**54**	I	pt2n0mx
60	male	**57**	II	pt3n0mx
61	male	**65**	II	pt3n0mx
62	male	**58**	II	t3n0mx
63	male	**65**	II	t4nomx
64	male	**67**	I	pt2n0mx

65	male	**55**	I	pt2n0mx
66	female	**77**	III	pt3n1mx
67	male	**54**	III	pt3n2amx
68	female	**55**	I	t2n0mx
69	female	**45**	non	non
70	male	**56**	I	pt2n0mx
71	male	**54**	III	pt2n2amx
72	male	**60**	non	non
73	female	**39**	non	non
74	male	**68**	II	t3nomx
75	female	**40**	III	pt3n1mx
76	male	**63**	III	pt3n1mx
77	female	**72**	III	pt4n1mx
78	female	**66**	II	mpt4n0mx
79	male	**69**	II	pt3n0mx
80	male	**65**	III	pt4n1mx
82	female	**55**	II	t4nomx
83	male	**66**	II	t3n0mx
84	female	**77**	II	t3nomx
85	male	**73**	II	pt3n0mx
86	male	**70**	III	pt3n2mx
87	female	**67**	II	pt3n0mx
88	male	**55**	IV	pt3n1m1
89	male	**70**	III	t4n1mx
90	male	**74**	II	pt(m)3n0mx
91	male	**64**	non	non
92	female	**52**	non	non
93	male	**27**	non	non
94	male	**63**	III	pt2n1cmx
95	female	**77**	III	pt3n1mx
96	female	**34**	non	non
97	male	**62**	III	pt3n2amx
98	female	**42**	non	non

99	female	**67**	non	non
100	male	**63**	III	pt3n2amx
101	female	**63**	III	Pt4n2mx
102	female	**36**	III	pt3n2bmx
103	male	**41**	non	non
104	male	**69**	II	pt3n0mx
105	female	**31**	III	pt3n2mx
106	male	**55**	III	pt4n1mx
107	female	**62**	II	pt4nomx
108	male	**63**	II	pt4nomx
109	male	**61**	II	pt4nomx
110	female	**62**	I	pt2n0mx
111	male	**81**	III	pt3n2mx

Table S2 Elisa raw data

Elisa.No.	OD-1	OD-2	Average OD	OD-blank	Concentration of HSPA8（ng/mL）
1	0.8484	0.9728	0.9106	0.8163	41.7908402
2	0.7939	0.7136	0.75375	0.65945	32.8933673
3	0.9686	0.7268	0.8477	0.7534	38.1460072
4	0.4963	0.5179	0.5071	0.4128	20.2072613
5	0.7333	0.7232	0.72825	0.63395	31.507597
6	0.7784	0.7537	0.76605	0.67175	33.5679058
7	0.6413	0.5626	0.60195	0.50765	24.8963935
8	0.3511	0.4921	0.4216	0.3273	16.1814138
9	0.7575	0.7079	0.7327	0.6384	31.7481959
10	0.6899	0.6018	0.64585	0.55155	27.1466983
11	0.7956	0.6107	0.70315	0.60885	30.1602719
12	0.7908	0.7126	0.7517	0.6574	32.7813305
13	0.5436	0.5595	0.55155	0.45725	22.3753951
14	0.9089	0.9516	0.93025	0.83595	42.9503176
15	0.4636	0.3498	0.4067	0.3124	15.4991589
16	0.6609	0.5035	0.5822	0.4879	23.9005527
17	0.5786	0.5307	0.55465	0.46035	22.5285323
18	0.5768	0.6916	0.6342	0.5399	26.5445746
19	0.5909	0.5648	0.57785	0.48355	23.6825933
20	0.5864	0.5599	0.57315	0.47885	23.4476554
21	0.5039	0.5534	0.52865	0.43435	21.2519525
22	0.7214	0.7087	0.71505	0.62075	30.7969758
23	0.5407	0.4283	0.4845	0.3902	19.124678
24	0.6079	0.5345	0.5712	0.4769	23.3503514
25	0.4725	0.4211	0.4468	0.3525	17.348305
26	0.4735	0.3691	0.4213	0.327	16.1676208
27	0.4525	0.4071	0.4298	0.3355	16.5593193
28	0.4767	0.4525	0.4646	0.3703	18.1824254
30	0.7283	0.7386	0.73345	0.63915	31.7887976
31	0.5125	0.4469	0.4797	0.3854	18.89646
32	0.4628	0.6128	0.5378	0.4435	21.6991924
33	0.5947	0.5436	0.56915	0.47485	23.2481652
34	0.7052	0.7445	0.72485	0.63055	31.3241197
35	0.6825	0.8094	0.74595	0.65165	32.46767
36	0.5875	0.5987	0.5931	0.4988	24.448888
37	0.6236	0.7301	0.67685	0.58255	28.7663359
38	0.494	0.5438	0.5189	0.4246	20.7777942
39	0.4555	0.438	0.44675	0.35245	17.3459735
40	0.3891	0.428	0.40855	0.31425	15.5835583
41	0.6142	0.7021	0.65815	0.53415	20.1245655
42	0.4919	0.509	0.50045	0.37645	14.1281094

43	0.5269	0.5456	0.53625	0.41225	15.4197968
44	0.6383	0.4184	0.52835	0.40435	15.1313492
45	0.5503	0.4989	0.5246	0.4006	14.9951077
46	0.4698	0.4858	0.4778	0.3538	13.3311252
47	0.3351	0.3902	0.36265	0.23865	9.51208708
49	0.5566	0.5055	0.53105	0.40705	15.2297137
50	0.5004	0.4225	0.46145	0.33745	12.7653962
51	0.6563	0.8064	0.73135	0.60735	23.1895028
52	0.3837	0.3921	0.3879	0.2639	10.3171352
53	0.3899	0.3773	0.3836	0.2596	10.1787824
54	0.5709	0.6075	0.5892	0.4652	17.4040201
55	0.3548	0.4243	0.38955	0.26555	10.3703618
56	0.4859	0.518	0.50195	0.37795	14.1814393
57	0.7954	0.8437	0.81955	0.69555	27.1360983
58	0.5237	0.4954	0.50955	0.38555	14.4527004
59	0.6849	0.6551	0.67	0.546	20.6081741
60	0.4851	0.5119	0.4985	0.3745	14.0588832
61	0.4479	0.5055	0.4767	0.3527	13.2928133
62	0.5155	0.4949	0.5052	0.3812	14.2972228
63	0.3504	0.3746	0.3625	0.2385	9.50735744
64	0.4351	0.3609	0.398	0.274	10.6441481
65	0.6145	0.6113	0.6129	0.4889	18.3213977
66	0.5788	0.5462	0.5625	0.4385	16.3923188
67	0.6767	0.6555	0.6661	0.5421	20.4484835
68	0.4887	0.4783	0.4835	0.3595	13.5302319
69	0.5713	0.6115	0.5914	0.4674	17.4884062
70	0.5257	0.4602	0.49295	0.36895	13.8624878
71	0.5116	0.4842	0.4979	0.3739	14.0376062
72	0.4913	0.5985	0.5449	0.4209	15.7378668
73	0.5718	0.5952	0.5835	0.4595	17.1861126
74	0.5866	0.5661	0.57635	0.45235	16.9142557
75	0.4804	0.4868	0.4836	0.3596	13.5337337
76	0.4486	0.3945	0.42155	0.29755	11.4178862
77	0.4188	0.3022	0.3605	0.2365	9.44435468
78	0.4993	0.5527	0.526	0.402	15.0459201
79	0.404	0.3482	0.3761	0.2521	9.93870701
80	0.4718	0.4586	0.4652	0.3412	12.8944469
82	0.5022	0.4345	0.46835	0.36	12.9680001
83	0.5874	0.5136	0.5505	0.44215	15.7829312
84	0.4034	0.6186	0.511	0.40265	14.4062918
85	0.3871	0.3472	0.36715	0.2588	9.74709583
86	0.4986	0.5736	0.5361	0.42775	15.2760275
87	0.4646	0.3501	0.40735	0.299	10.9949888
88	0.5271	0.6356	0.58135	0.473	16.8886709

89	0.5158	0.5714	0.5436	0.43525	15.539313
90	0.1891	0.1937	0.1914	0.08305	4.74911752
91	0.3919	0.5386	0.46525	0.3569	12.8653697
92	0.5196	0.4108	0.4652	0.35685	12.8637165
93	0.8935	0.6318	0.76265	0.6543	23.9586129
94	0.5112	0.527	0.5191	0.41075	14.6850566
95	0.4524	0.5591	0.50575	0.3974	14.226575
96	0.5296	0.6986	0.6141	0.50575	18.0925108
97	0.5565	0.5135	0.535	0.42665	15.2375447
98	0.0994	0.1073	0.10335	-0.005	2.50536779
99	0.4857	0.463	0.47435	0.366	13.1673693
100	0.4045	0.3473	0.3759	0.26755	10.0152486
101	0.4321	0.487	0.45955	0.3512	12.6773304
102	0.4999	0.5011	0.5005	0.39215	14.0476132
103	0.7067	0.7228	0.71475	0.6064	21.9924778
104	0.9608	0.8441	0.90245	0.7941	30.1262276
105	0.4517	0.3494	0.40055	0.2922	10.7810255
106	0.1895	0.1668	0.17815	0.0698	4.40088238
107	0.8135	0.8747	0.8441	0.73575	27.4723673
108	0.4545	0.4778	0.46615	0.3578	12.8951392
109	0.5169	0.4047	0.4608	0.35245	12.7184931
110	0.7885	0.6539	0.7212	0.61285	22.2530027
111	0.4389	0.5143	0.4766	0.36825	13.2423815

Table S3. HSPA8-score

Elisa No.	Stage	concentration-plasma(ug/ul)	amount-plasma (10ul)	concentration-HSPA8(ng/ml)	HSPA8-score
1	I	32.26	322.58	41.79	0.13
2	I	49.06	490.63	32.89	0.07
3	I	40.65	406.50	38.15	0.09
4	II	60.76	607.62	20.21	0.03
5	II	49.08	490.80	31.51	0.06
6	III	74.35	743.51	33.57	0.05
7	III	32.26	322.58	24.90	0.08
8	non	113.09	1130.87	16.18	0.01
9	non	80.83	808.35	31.75	0.04
10	I	27.86	278.55	27.15	0.10
11	non	80.37	803.74	30.16	0.04
12	non	76.23	762.27	32.78	0.04
13	III	68.21	682.10	22.38	0.03
14	II	66.35	663.46	42.95	0.06
15	I	62.15	621.52	15.50	0.02
16	III	52.47	524.68	23.90	0.05
17	III	50.52	505.16	22.53	0.04
18	II	46.72	467.21	26.54	0.06
19	I	65.05	650.45	23.68	0.04
20	III	58.65	586.48	23.45	0.04
21	II	51.38	513.84	21.25	0.04
22	III	72.85	728.52	30.80	0.04
23	IV	73.83	738.28	19.12	0.03
24	II	75.45	754.54	23.35	0.03
25	III	67.21	672.14	17.35	0.03
26	III	64.18	641.78	16.17	0.03
27	III	67.54	675.39	16.56	0.02
28	I	69.71	697.08	18.18	0.03
30	I	76.10	761.05	31.79	0.04
31	II	80.88	808.75	18.90	0.02
32	II	68.30	682.98	21.70	0.03
33	III	85.32	853.21	23.25	0.03
34	III	62.99	629.85	31.32	0.05
35	III	67.43	674.31	32.47	0.05
36	III	70.57	705.75	24.45	0.03
37	I	53.23	532.27	28.77	0.05
38	IV	68.08	680.81	20.78	0.03
39	III	15.50	154.95	17.35	0.11

40	III	75.13	751.29	15.58	0.02
41	IV	64.72	647.20	20.12	0.03
42	III	65.26	652.62	14.13	0.02
43	I	59.78	597.82	15.42	0.03
44	III	78.60	785.98	15.13	0.02
45	II	62.51	625.09	15.00	0.02
46	II	52.58	525.76	13.33	0.03
47	II	86.62	866.22	9.51	0.01
49	II	46.07	460.71	15.23	0.03
50	II	62.88	628.77	12.77	0.02
51	II	59.08	590.82	23.19	0.04
52	II	77.19	771.89	10.32	0.01
53	III	56.70	566.97	10.18	0.02
54	III	72.96	729.60	17.40	0.02
55	III	82.50	825.02	10.37	0.01
56	II	77.51	775.14	14.18	0.02
57	III	79.68	796.83	27.14	0.03
58	II	63.64	636.36	14.45	0.02
59	I	61.74	617.40	20.61	0.03
60	II	64.83	648.28	14.06	0.02
61	II	64.07	640.69	13.29	0.02
62	II	69.71	697.08	14.30	0.02
63	II	40.32	403.24	9.51	0.02
64	I	67.76	677.56	10.64	0.02
65	I	86.30	862.97	18.32	0.02
66	III	20.92	209.16	16.39	0.08
67	III	93.56	935.61	20.45	0.02
68	I	65.05	650.45	13.53	0.02
69	non	53.77	537.69	17.49	0.03
70	I	54.74	547.45	13.86	0.03
71	III	21.78	217.84	14.04	0.06
72	non	63.31	633.11	15.74	0.02
73	non	50.08	500.83	17.19	0.03
74	II	63.42	634.19	16.91	0.03
75	III	54.31	543.11	13.53	0.02
76	III	85.10	851.04	11.42	0.01
77	III	42.71	427.10	9.44	0.02
78	II	47.26	472.64	15.05	0.03
79	II	77.41	774.06	9.94	0.01
80	III	92.26	922.60	12.89	0.01
82	II	62.88	628.77	12.97	0.02
83	II	50.62	506.25	15.78	0.03
84	II	60.27	602.75	14.41	0.02

85	II	44.34	443.36	9.75	0.02
86	III	68.51	685.15	15.28	0.02
87	II	52.68	526.85	10.99	0.02
88	IV	61.81	618.14	16.89	0.03
89	III	67.86	678.64	15.54	0.02
90	II	70.25	702.50	4.75	0.01
91	non	76.97	769.72	12.87	0.02
92	non	82.49	824.91	12.86	0.02
93	non	73.63	736.27	23.96	0.03
94	III	60.86	608.65	14.69	0.02
95	III	20.70	206.99	14.23	0.07
96	non	79.47	794.72	18.09	0.02
97	III	71.61	716.10	15.24	0.02
98	non	73.92	739.24	2.51	0.00
99	non	99.73	997.26	13.17	0.01
100	III	39.13	391.32	10.02	0.03
101	III	43.76	437.60	12.68	0.03
102	III	43.58	435.77	14.05	0.03
103	non	89.51	895.05	21.99	0.02
104	II	68.78	687.79	30.13	0.04
105	III	59.84	598.41	10.78	0.02
106	III	55.72	557.21	4.40	0.01
107	II	90.42	904.24	27.47	0.03
108	II	55.15	551.50	12.90	0.02
109	II	81.03	810.33	12.72	0.02
110	I	59.58	595.75	22.25	0.04
111	III	57.69	576.85	13.24	0.02

Table S4. Confusion matrices and diagnostic performance of HSPA8-score-based random forest classification.

Comparison	TP / FN / FP / TN	Sensitivity	Specificity	PPV	NPV	Accuracy
Matched Set: Stage I vs non-tumor (n=18)	5 / 3 / 3 / 7	62.50%	70.00%	62.50%	70.00%	66.67%
Expanded Set: Stage I vs non-tumor (n=30)	10 / 6 / 7 / 7	62.50%	50.00%	58.82%	53.85%	56.67%
Matched Set: Stage I vs Stages II–III (n=22)	5 / 3 / 3 / 11	62.50%	78.57%	62.50%	78.57%	72.73%
Expanded Set: Stage I vs Stages II–IV (n=94)	4 / 12 / 12 / 66	25.00%	84.62%	25.00%	84.62%	74.47%

Table S5. Correlations of ELISA/Protein abundance with Age and Sex

measurement	covariate	n	rho	p	q_BH
HSPA8(ng/ml)	age	108	-0.075006225	0.440407683	0.815319002
HSPA8(ng/ml)	sex	108	-0.0494226	0.611489252	0.815319002
HSPA8-score	age	108	-0.000953489	0.992185924	0.992185924
HSPA8-score	sex	108	-0.079974752	0.410638837	0.815319002
ALDOA plasma_abundance	age	34	-0.175216394	0.321608268	0.556014375
ALDOA plasma_abundance	sex	34	0.161209125	0.362390303	0.556014375
HSPA8 plasma_abundance	age	34	-0.092241735	0.603867175	0.72464061
HSPA8 plasma_abundance	sex	34	0.272040398	0.119603356	0.556014375
PDLIM1 plasma_abundance	age	34	-0.158481213	0.37067625	0.556014375
PDLIM1 plasma_abundance	sex	34	0.021426863	0.904261833	0.904261833
